# Foods to Avoid While Breastfeeding? Experiences and Opinions of Polish Mothers and Healthcare Providers

**DOI:** 10.3390/nu12061644

**Published:** 2020-06-02

**Authors:** Karolina Karcz, Izabela Lehman, Barbara Królak-Olejnik

**Affiliations:** Department and Clinic of Neonatology, Wroclaw Medical University, Borowska 213, 50-556 Wroclaw, Poland; izabela.lehman@student.umed.wroc.pl (I.L.); barbara.krolak-olejnik@umed.wroc.pl (B.K.-O.)

**Keywords:** breastfeeding, diet, health care surveys

## Abstract

Popular beliefs regarding a mother’s diet during lactation have a significant impact on breastfeeding practices among mothers, as well on breastfeeding counseling among healthcare providers worldwide. The objective of this study was to assess mothers’ and medical professionals’ knowledge and opinions on the “lactating mother’s diet”. An electronic survey, prepared in Polish, was administered to healthcare providers, as well as mothers who have breastfed a child. The chi-square test, logistic regression, and Mann Whitney U test were used for statistical calculations. Out of a total of 1180 responses received, 1159 were analyzed, and 21 were excluded because they did not meet the inclusion criteria. The survey was completed by 407 (35%) medical healthcare providers and 752 (65%) lactating mothers in non-medical professions. In total, the study included 1074 mothers who have breastfed a child, and 29.14% of them reported that they eliminated certain foods from their diet when breastfeeding. There was no statistically significant difference in the responses received from mothers and medical staff providing maternal care (for each of 17 products, e.g., steak tartare, sushi, legumes, dairy products, *p* > 0.05 by the Mann-Whitney test). However, a logistic regression revealed some significant correlations with other variables (e.g., duration of lactation). The respondents revealed an appropriate level of knowledge on nutrition during lactation and the majority of participants neither adhered to nor recommended a prophylactic elimination diet. Among other evaluated factors, the experience of following an elimination diet affected respondents’ knowledge of nutrition during breastfeeding. Both mothers and healthcare providers require good nutritional education.

## 1. Introduction

Human milk provides optimal nutrition for infants. Thus, it constitutes a first choice for feeding [1,2]. On the basis of numerous short- and long-term benefits for both mothers and infants, multiple organizations, such as the American Academy of Pediatrics (AAP) [3], the American College of Obstetricians and Gynecologists (ACOG) [4], and the World Health Organization (WHO) [5], support the recommendation of exclusive breastfeeding for at least the first 6 months of life, with its continuation alongside weaning. As far as the impact on several health conditions is concerned, according to the current state of knowledge, exclusive breastfeeding for 3 to 4 months reduces the risk of atopic diseases, including eczema and asthma. Moreover, feeding with human milk while introducing complementary foods might decrease the incidence of food allergies [6].

On the other hand, exclusively breastfed infants might present symptoms suggesting manifestations of allergic reactions to food components. The concentration of antigens or endogenous allergenic proteins in breast milk can be high enough to trigger an allergic reaction. Nevertheless, the diagnosis of food allergy in exclusively breastfed infants is challenging and requires conscientious consideration, as well as the exclusion of other conditions [7]. In fact, the first treatment of food allergy is the cessation of allergen exposure, which in practice means an elimination diet in lactating mothers. However, there is no evidence that excluding some products from the diet of pregnant or lactating mothers as a preventive measure can protect her offspring from developing a sensitization to allergens or any atopic disease [6,8].

Despite the scientific evidence, the popular beliefs regarding the mother’s diet during lactation have a significant impact on breastfeeding practices in society, as well breastfeeding counseling among healthcare providers worldwide. Mothers are constantly reinforced in their conviction that their breast milk can be harmful for infants unless they change their dietary habits and stop consuming certain products when breastfeeding. The authors of this work have often experienced lactating women consulting their clinic to ask whether dietary restrictions are required and what they should eat. The most common misconceptions concern not only the prevention of atopic diseases, but also human milk quality and quantity, the effectiveness of lactation, milk-borne diseases and infants’ general condition and development, all of which are attributable to the consumption of certain products [9,10,11].

The period of lactation imposes the need for an additional energetic and nutrient supply for mothers [12]. The dietary restrictions not only might affect the effectiveness of breastfeeding, but they can be detrimental for mothers’ nutritional status and well-being. Constant anxiety about infants’ health and the further elimination of potentially “harmful” products from the diet are factors which contribute to the decision to stop breastfeeding [13].

## 2. Aim of the Study

The objective of this study was to assess mothers’ (representing the general public), and medical professionals’ knowledge and opinions related to the issue of diet during lactation and the consumption of particular products.

## 3. Materials and Methods 

The electronic survey was conducted in January and February 2019 in Poland among native inhabitants of Poland. The target group of the study included: (1) mothers who have breastfed a child (generally healthy and born full-term), regardless of the duration of lactation, including expressed breast milk with bottle feeding, for example, as representatives of Polish society; and (2) healthcare providers, regardless of age, sex, and parity. The questionnaire (Supplementary Materials File S1) used in the study was prepared by the authors, in a Polish language version only. It consisted of three parts: (1) questions regarding whether a lactating mother can eat or drink certain foods; (2) questions concerning popular myths related to nutrition when breastfeeding a child and referring to the knowledge of breast milk composition; and (3) demographic data and respondents’ experience with an elimination diet. In this study, the first part of the results is presented, referring to the opinions regarding the consumption of particular products during lactation. The list of foods included in the study was compiled from information found online (e.g., discussion groups and forums, parenting blogs) or received directly from lactating mothers consulting the authors’ clinic.

The survey was distributed in an electronic form, mainly using randomly selected internet discussion groups and forums: (1) related to the topics of parenthood, or breastfeeding, available mainly on Facebook; or (2) established for medical professionals (doctors, midwives, nurses, lactation consultants, etc.). To ensure participants’ comprehension of the questionnaire, face-to-face interviews with a convenient sample (*n* = 10) were conducted prior to the start of the study. Provided that the respondents agreed to participate in the study through direct contact, they were provided with a link to the questionnaire. The participation in the study was fully voluntary. The responses were collected anonymously and were coded automatically.

Prior to the study’s commencement, formal permission was obtained from the Bioethics Committee at the Medical University in Wroclaw (No. KB 519/19).

Formal analysis of the results was performed using the chi-square test (to test for differences in the correct answer rate categorized by the place of residence), logistic regression (to assess a relationship between predictor variables and respondent’s answers, categorized as correct and incorrect) and the Mann-Whitney U test (to compare answers between study groups). Calculations were made in Microsoft Excel for Office 365 (Microsoft, Redmond, WA, USA), STATISTICA 13.3 (StatSoft, Inc., Tulsa, OK, USA) and R version 3.6.2 (R Core Team, 2013. R Foundation for Statistical Computing, Vienna, Austria. http://www.R-project.org/).

## 4. Results

### 4.1. Data

**Figure 1:**
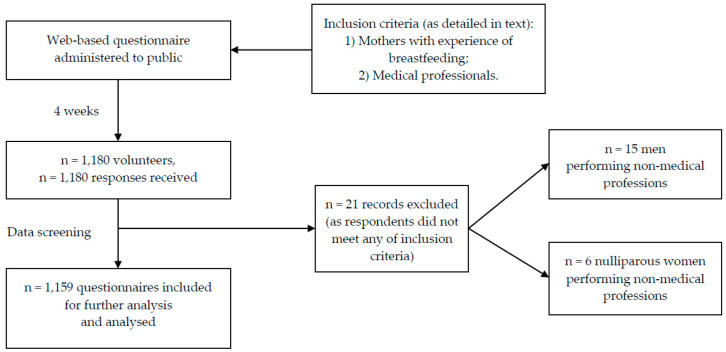
Flowchart on study group recruitment steps.

Questionnaire data were provided by 1180 respondents, all of whom were native Polish speakers. As 21 respondents did not meet the basic criteria of participation, as neither a medical professional, nor a mother who had ever fed a baby with her own breast milk, for the purpose of the formal analysis, 1159 questionnaires were included (Figure 1).

### 4.2. Characteristics of the Study Group

The group of respondents comprised: (1) 407 individuals carrying out a medical profession, 10 men and 397 women, including 322 mothers who delivered their babies at term and reported a history of breastfeeding; (2) mothers with any experience in breastfeeding, pursuing professions outside the healthcare sector (*n* = 752). The respondents differed in terms of age, level of education, place of residence, parity, history of breastfeeding and experience in practicing an elimination diet during lactation. The detailed characteristics of the study group are provided in Table 1.

### 4.3. Foods to Avoid While Breastfeeding

**Figure 2:**
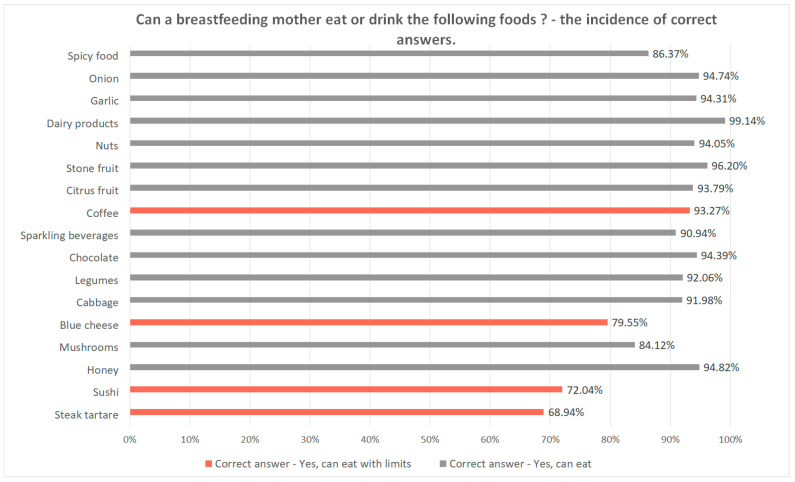
Can a lactating mother eat or drink the following products: incidence of correct answers for each of the 17 products included in the questionnaire. The bars on the chart referring to foods that can be eaten with limits are colored orange, and the bars referring to foods that do not need any prophylactic restriction are colored gray.

The questions regarding whether the consumption of certain products is allowed in the lactation period referred to the most confusing food, as there are a variety of contrary opinions and various myths related to including them in the daily diet of lactating mothers. The list of products commonly advised to be avoided, as a preventive measure, was based on information found on the internet, leaflets for patients or provided directly by mothers and medical personnel. It included 17 items: steak tartare, sushi, honey, mushrooms, cheese (including blue cheese), cabbage, legumes, chocolate, sparkling beverages (including sparkling water), coffee, citrus fruits, stone fruits, nuts, dairy products, garlic, onion, and spicy food. The incidence of correct answers among all the respondents ranged from 68.94% to 99.14% and was higher than 90% in 12 of 17 items (70.59%) (Figure 2). The rate of correct answers differed by place of residence only for two products: honey (χ2 (2, N = 1159) = 8.138, *p* = 0.017) and spicy food (χ2 (2, N = 1159) = 6.833, *p* = 0.033). No statistically significant difference in answers was found between healthcare professionals and mothers performing non-medical professions (for each of the 17 products, *p* > 0.05 by Mann-Whitney test).

In further analysis, the respondents’ answers referring to each of the 17 products were correlated with the following variables: parity, gender, duration of breastfeeding the first child, following the elimination diet when breastfeeding the child, feeding the infant with commercial formula instead of breastfeeding because of an elimination diet, pursuing a medical profession, and following an elimination diet due to a doctor’s advice. Logistic regression calculations revealed no correlations with feeding the infant with commercial formula instead of breastfeeding because of an elimination diet, nor pursuing a medical profession. Only with regard to “dairy products” did answers differ depending on respondents’ gender. The answers concerning the following 16 products were correlated with the other variables to a different extent. The results are summarized in Table 2.

### 4.4. Elimination Diet According to Experience of Mothers and Medical Professionals

Among the respondents, 29.14% of all mothers indicated that they had ever followed an elimination diet while breastfeeding their children. In the most cases, this practice resulted from following a doctor’s advice (71.25%, *n* = 223), peer pressure—e.g., received from family, friends (20.77%, *n* = 65), or other reasons (7.99%, *n* = 25), including the mother’s anxiety that her diet might have negatively affected her milk and the breastfed child. Within the aforementioned group, 95.52% (*n* = 217) of mothers were advised to eliminate dairy products due to the suspicion of an allergy to milk proteins in the infant. Among the respondents, 16.93% (*n* = 53) of mothers decided to stop breastfeeding and start feeding their infants with commercial milk formula because of dietary restrictions. As far as an elimination diet being considered as a preventive measure, 61 out of 407 (~15%) healthcare professionals admitted that they had recommended this practice to their breastfeeding patients.

## 5. Discussion

Our experience from clinical practice indicates that the myths concerning the “lactating mother’s diet” and conviction regarding the advantages of prophylactic dietary restrictions are still widely present in Polish society. Even though the respondents presented a good level of knowledge on the allowance of consumption of several products during lactation, a portion of the respondents, i.e., about 10–30% depending on the product, contested the possibility of including some foods in a daily diet when breastfeeding. The results of the survey revealed that multiparas and mothers who had the possibility to breastfeed their first child were more likely to give correct answers. Mothers who followed an elimination diet more often answered incorrectly. However, the results and correct answers rate improved when a dietary restriction was started due to a doctor’s advice.

The reasons for dietary restrictions and planning a “lactating mother’s diet” varied depending on factors including the conviction of their role in the prophylaxis of allergies, peer pressure, cultural background, or the attempt to manage or clarify symptoms observed in infants. With regard to the aforementioned issues, we will refer to the available literature.

As has been already mentioned, a mother’s “diet mistakes” are believed to be a main cause of infant colic. In fact, this condition improves over time, regardless of intervention. On the basis of the authors’ previous clinical practice, most often, mothers believe that bloating or colic pain in their offspring might be caused by the consumption of legumes, cabbage, onion, garlic or sparkling beverages, as the gas molecules transfer to the breast milk and irritate the infant’s digestive tract. The results of the available studies remain divergent: some literature indicates the essential role of particular dietary restrictions while breastfeeding in reducing the manifestation of colic symptoms in infants, at the same time, a number of papers underline the ineffectiveness of any modification in the mother’s or infant’s nutrition [14,15]. For this reason, no recommendation on dietary modifications in the treatment of infant colic has been made [15].

As far as the prevention of atopy and other allergies is concerned, the available data remain ambiguous. On the basis of current evidence, there is no premise to support prophylactic maternal dietary restrictions during the lactation period, or in pregnancy. Nevertheless, the identification and elimination of allergens from the diet might be supportive for the treatment of children who have developed atopic diseases [6,8,13].

Unfortunately, a number of mothers worldwide still attribute their infant’s fussiness and gastrointestinal symptoms to feeding and “diet mistakes”, leading to further dietary restrictions as a preventive measure. The list of the most commonly suspected products includes coffee, sparkling beverages, legumes, citrus fruits, dairy products, chocolate, cabbage, garlic, onion and spicy food [16]. This belief is often supported by family and friends in the mother’s surroundings [10,16], which has been reflected in the results of our study, and this might have a cultural background [11]. This issue is consistent with observations from the authors’ clinical experience. For example, an insufficient quantity of human milk is attributable to various “diet mistakes”, for example, mothers are often told they should drink large portions of still water or Bavarian tea to increase milk supply. Moreover, it is believed that milk stasis in the lactiferous duct or low milk secretion result from the consumption of stone fruits, as the stone can cause an occlusion of the lactiferous ducts. It is important to emphasize that, taking peer pressure into consideration, the conviction that dietary habits need to be changed as a preventive measure and restrictions on food may not only cause stress and disturb successful breastfeeding, resulting in its short duration, but may adversely affect a mother’s health condition and her micronutrient body reserves [17].

Another controversial practice is the consumption of raw meat and fish, for example, steak tartare, sushi, and unpasteurized milk or cheese, including blue cheese. During pregnancy, the consumption of these products is contraindicated, but during lactation the limitations are less strict. Provided that the meat is fresh and comes from a verified supplier, it can be eaten raw, with the exception of poultry and venison. Sushi should be prepared with fresh fish and it is recommended to choose species which are less likely to accumulate mercury, such as the small and non-predatory varieties, as well as the other seafood. The same rules concern unpasteurized products, they should be fresh, purchased from a verified supplier and should not exceed the best-before date. Reasonable choices are essential, as the consumption of the aforementioned products is connected with the risk of food-borne infections in mothers. However, apart from rare cases of septicemia, they do not pose a significant threat to the breastfed infant [10,17,18].

In addition, breastfeeding is considered to be an essential factor contributing to the promotion of a healthy diet, it has a leading role in modulating the development of flavor preferences. Infants exposed to various scents and flavors in breast milk, which stem from their mothers’ diets, are more likely to accept them during complementary feeding and in later childhood. Moreover, children who were breastfed in infancy are believed to be more willing to eat vegetables than formula-fed infants [19,20].

The issue of nutrition during the lactation period, including dietary choices and apprehension about consuming particular foods, seems to be a relevant matter worldwide. Based on the available literature, opinions on the link between mother’s nutrition and infants’ behavior have already been investigated in Canada [16], Asia [10,11,21], Mexico [22], and the USA [23]. Misinformation related to mainly animal-source foods or fruits and vegetables lead to women feeling obliged to deal with dietary restrictions, believing this practice to bring health benefits for their offspring [10,11,16,21,22,24,25]. Nevertheless, different degrees of elimination of various products from the diet make mothers vulnerable to qualitative malnutrition, resulting especially from deprivation of micronutrients [18,21]. As a result of nutritional stress, the quality of life is diminished. Additionally, following an elimination diet is a factor affecting the length of breastfeeding [1,23,24]. In fact, knowledge gaps, commitment to tradition, and peer pressure make lactating mothers follow the practice of myths and taboos and to consequently develop learned and observed habits [10,11,16,24,25]. In this paper, our aim was to assess the knowledge on nutrition of breastfeeding mothers in Polish society, with a special focus on the comparison between healthcare providers and patients and emphasis on the presence of particular products in daily diets. In similar publications of Polish authors, the general knowledge of lactation, including popular myths and nutrition, was investigated among mothers [24] and healthcare providers [25]. The results of the aforementioned studies are comparable to the results of this survey, thus, further education in the area of lactation is needed, and knowledge of nutrition and reliable counseling are crucial for long and successful breastfeeding.

### Recommendations

Breastfeeding is a fully physiological condition, therefore, a “healthy breastfeeding mother’s diet” should be prepared in accordance with the current recommendations included in the nutrition pyramid and include a variety of products. This is essential as it might allow the lactating mothers to cover their increased daily requirements for nutrients, vitamins, and microelements [12,26].

General rules of nutrition during lactation should be followed:

1. Routine elimination diets, e.g., to prevent allergies in a child, are not recommended [7,8,13,14,15].
2. The choice of natural, low-processed products of good quality is recommended [18,26].
3. Foods containing preservatives, artificial additives, and trans fatty acids should be avoided [26].
4. The consumption of products with a high sugar content, such as sweets or large amounts of fruit juice, is not recommended [26].
5. Energy demands during the first 6 months of lactation increase by approximately 500 kcal per day, therefore, a breastfeeding mother should consume several meals daily [26,27].
6. Overeating or eating “for two” is not advised, it is enough to satisfy hunger [26].
7. Breastfeeding mothers should supply an additional 21 g of proteins per day in the initial period of lactation and 14 g in the next months of breastfeeding [12,18,26].
8. The daily supplementation of 100–200 mg of docosahexaenoic acid (DHA) or regular consumption of two servings of fish per week enables an adequate DHA content in breast milk to be reached and provides the infant with a proper amount of polyunsaturated fatty acids [3,18,28].
9. Breastfeeding requires the provision of iron at doses of 11 mg per day in the first months of lactation, and 18 mg per day after the resumption of menstruation [18,29].
10. Adequate iodine intake (200 µg daily) ensures an adequate iodine content in breast milk (approximately 100–150 µg/100 mL) [30].
11. As the amount of calcium secreted in breast milk is 150–300 mg per day and depends on mother’s bone mineralization and urinary calcium excretion, the recommended daily calcium intake in the mother’s diet is 1000 mg [18,31]. The main dietary sources of calcium include milk and dairy products, followed by cereals and vegetables [12,18,26].
12. Supplementation of folic acid at a dose of 500 ug per day, as well as the consumption of natural sources of folates (e.g., green leafy vegetables, oranges, cereals, and offal), is recommended [12,32].
13. Vitamin D should be administered under the control of 25(OH)-D serum levels, with an optimal range of 30–50 ng/mL. If it is not possible to determine the concentration of 25(OH)-D, 2000 IU of vitamin D should be administered daily throughout lactation. High amounts of vitamin D are contained in cod liver oil and fatty fish, such as herring and salmon [18,33].

## 6. Limitations

The foremost limitation of the survey is its reliance on answers self-provided by respondents. There is no possibility of verifying the accuracy of the data, nor the identity of the respondent. To minimize the risk of ineligibility, the questionnaire provided questions referring to the basic inclusion criteria, e.g., category of practiced profession (medical or non-medical), gender, and parity. Based on the received answers, questionnaires completed by 21 respondents were excluded from further analysis.

Another limitation is that a truly random sample of participants was impossible to obtain, the survey was responded to mostly by mothers and medical professionals who were active on randomly selected discussion groups and forums. For this reason, the study group should not be considered as fully representative of the Polish population.

## 7. Conclusions

The nutrition of women during lactation is a controversial topic. In the past, mother’s nutrition was attributed to all adverse symptoms in the infant, however, our knowledge is changing, meaning that some theories are being scientifically confirmed while others are refuted. The lack of current knowledge causes the reproduction of many harmful myths. 

The topic of the “lactating mother’s diet” is still an important issue worldwide, as the traditional beliefs concerning the nutrition of breastfeeding women, its impact on infant’s health and need of dietary restrictions affect the practice of breastfeeding. This seems to be confirmed by the results of the survey: almost 30% of mothers were advised to start dietary restrictions, not only for medical reasons, leading over 16% of them to stop breastfeeding. In addition, among other evaluated factors, the experience of following elimination diet affected mothers’ knowledge of nutrition during breastfeeding. However, the majority of respondents correctly recognized the questioned foods as generally permitted when lactating. 

Although the level of knowledge is improving and adherence to taboos is diminishing, both mothers and healthcare providers require good nutritional education. Responsible nutrition should be based on reasonable choices, whenever dietary restrictions need to be introduced due to medical indications, the mother’s diet should be well-planned and cover all nutritional demands.

## Figures and Tables

**Table 1:** Characteristics of respondents (*n* = 1159).

Variable	Number of Respondents (%)
**Age in years**	
<20	3 (0.26%)
20–29	490 (42.28%)
30–39	581 (50.13%)
≥40	85 (7.33%)
**Gender**	
Female	1149 (99.14%)
Male	10 (0.86%)
**Place of residence**	
Village	213 (18.38%)
Cities < 100,000 residents	301 (25.97%)
Cities > 100,000 residents	645 (55.65%)
**Profession**	
Medical	407 (35.12%)
Non-medical	752 (64.88%)
**Parity**	
0	77 (6.64%)
1	586 (50.56%)
2	426 (36.76%)
3	67 (5.78%)
4 or more	3 (0.26%)
**Duration of first child’s breastfeeding (months)**	
0–3	172 (14.84%)
4–5	94 (8.11%)
6–12	327 (28.21%)
13–24	363 (31.32%)
More than 24	118 (10.18%)
n/a	85 (7.33%)

Abbreviations: n/a—non applicable.

**Table 2:** Statistically significant factors with an influence on the opinions regarding whether a mother is allowed to consume particular foods while breastfeeding. Multiple regression analysis, *p* < 0.05.

Item	Variables						
	Parity	Gender	Duration of Breastfeeding the 1st Child	Following the Elimination Diet When Breastfeeding the Child	Feeding the Infant with Commercial Formula Instead of Breastfeeding for the Reason of Elimination Diet	Practicing a Medical Profession	Following an Elimination Diet Due to a Doctor’s Advice
	B; OR (95% CI)	B; OR (95% CI)	B; OR (95% CI)	B; OR (95% CI)	B; OR (95% CI)	B; OR (95% CI)	B; OR (95% CI)
Steak tartare	−0.246; 0.78 (0.66, 0.93)	n/s	0.026; 1.03 (1.01, 1.04)	0.700; 0.5 (0.38, 0.65)	n/s	n/s	−0.416; 0.66 (0.49, 0.89)
Sushi	−0.213; 0.81 (0.68, 0.97)	n/s	0.031; 1.03 (1.02, 1.05)	−0.691; 0.5 (0.38, 0.66)	n/s	n/s	−0.335; 0.72 (0.52, 0.98)
Honey	n/s	n/s	0.036; 1.04 (1, 1.07)	−0.916; 0.4 (0.24, 0.68)	n/s	n/s	n/s
Mushrooms	−0.311; 0.73 (0.59, 0.91)	n/s	0.0304; 1.03 (1.01, 1.05)	−1.034; 0.36 (0.26, 0.49)	n/s	n/s	n/s
Cheese	−0.252; 0.78 (0.64, 0.95)	n/s	0.040; 1.04 (1.02, 1.06)	−0.911; 0.4 (0.3, 0.54)	n/s	n/s	−0.426; 0.65 (0.46, 0.92)
Cabbage	n/s	n/s	0.051; 1.05 (1.02, 1.08)	−1.561; 0.21 (0.14, 0.33)	n/s	n/s	−0.477; 0.62 (0.38, 1.01)
Legumes	n/s	n/s	0.043; 1.04 (1.01, 1.07)	−1.692; 0.18 (0.12, 0.29)	n/s	n/s	−0.55; 0.57 (0.36, 0.93)
Chocolate	n/s	n/s	0.053; 1.05 (1.02, 1.09)	−1.433; 0.24 (0.14, 0.4)	n/s	n/s	n/s
Sparkling beverages	−0.389; 0.68 (0.51, 0.89)	n/s	0.047; 1.05 (1.02, 1.08)	−0.919; 0.4 (0.27, 0.6)	n/s	n/s	n/s
Coffee	n/s	n/s	0.044; 1.04 (1.01, 1.08)	−1.193; 0.3 (0.19, 0.48)	n/s	n/s	−0.744; 0.48 (0.29, 0.78)
Citrus fruits	n/s	n/s	0.066; 1.07 (1.03, 1.11)	−1.693; 0.18 (0.11, 0.3)	n/s	n/s	n/s
Stone fruits	−0.461; 0.63 (0.42, 0.95)	n/s	0.045; 1.05 (1.01, 1.09)	−1.733; 0.18 (0.09, 0.33)	n/s	n/s	n/s
Nuts	n/s	n/s	0.043; 1.04 (1.01, 1.08)	−1.819; 0.16 (0.1, 0.27)	n/s	n/s	−0.939; 0.39 (0.23, 0.65)
Dairy products	n/s	2.644; 14.07 (1.61, 122.97)	n/s	n/s	n/s	n/s	n/s
Garlic	n/s	n/s	0.107; 1.11 (1.07, 1.16)	−1.813; 0.16 (0.1, 0.28)	n/s	n/s	−0.566; 0.57 (0.33, 0.99)
Onion	n/s	n/s	0.084; 1.09 (1.04, 1.13)	−1.99; 0.14 (0.08, 0.24)	n/s	n/s	n/s
Spicy food	n/s	n/s	0.061; 1.06 (1.04, 1.09)	−1.187; 0.31 (0.22, 0.43)	n/s	n/s	n/s

Abbreviations: B—estimate coefficient; OR—odds ratio; CI—confidence interval; n/s—non-significant.

## Supplementary Materials

The following are available online at https://www.mdpi.com/2072-6643/12/6/1644/s1, File S1: Questionnaire.
