# Supplementary material for: Foods to Avoid While Breastfeeding? Experiences and Opinions of Polish Mothers and Healthcare Providers
Source: Nutrients. 2020 Jun 2;12(6):1644. doi: 10.3390/nu12061644 (PMC7352950; doi:10.3390/nu12061644)
Supplement: Supplementary file 1 [file nutrients-12-01644-s001.pdf]

Dieta matki karmiącej piersią - fakty czy mity?

Szanowni Państwo!

Jesteśmy doktorantkami w Klinice Neonatologii Uniwersytetu Medycznego we Wrocławiu. Zwracamy się z prośbą o wypełnienie kwestionariusza ankietowego, przygotowanego w ramach badania dotyczącego diety matki karmiącej piersią w Polsce. Adresatami ankiety są Panie, które kiedykolwiek karmiły dziecko piersią (dopuszcza się karmienie mlekiem własnym przy pomocy np. butelki) - prosimy, aby ankietę wypełniły Panie, które urodziły dziecko po ukończonym 37 tygodniu ciąży; oraz personel medyczny (bez względu na płeć i dzietność). Prosimy

Niniejsza ankieta jest w pełni anonimowa. Uzyskane wyniki zostaną wykorzystane do przygotowania opracowania naukowego .

Wypełnienie ankiety jest równoznaczne ze zgodą na udział w badaniu.

Bardzo dziękujemy za poświęcenie Państwa czasu!
*Wymagane

1. *Zaznacz tylko jedną odpowiedź.*

   ◯ Opcja 1

2. Czy matka karmiąca piersią może jeść niżej wymienione produkty/ potrawy? *

   Zaznacz tylko jedną odpowiedź w rzędzie.

	Tak	Nie
Tatar	◯	◯
Sushi	◯	◯
Miód	◯	◯
Grzyby	◯	◯
Sery pleśniowe	◯	◯
Kapusta	◯	◯
Nasiona strączkowe, np. groch, fasola	◯	◯
Czekolada	◯	◯
Woda i napoje gazowane	◯	◯
Kawa	◯	◯
Cytrusy	◯	◯
Owoce pestkowe	◯	◯
Orzechy	◯	◯
Nabiał	◯	◯
Czosnek	◯	◯
Cebula	◯	◯
Pikantne przyprawy	◯	◯

3. Proszę odnieść się do poniższych stwierdzeń zgodnie z własnymi przekonaniami: *

Zaznacz tylko jedną odpowiedź w rzędzie.

	Prawda	Fałsz
Bawarka (herbata z mlekiem) zwiększa laktację	◯	◯
Picie dużej ilości wody zwiększa produkcję mleka	◯	◯
Dziecko karmione piersią płacze, bo matka niewłaściwie się odżywia	◯	◯
Ostre przyprawy w pożywieniu zmieniają smak mleka matki	◯	◯
Dieta matki karmiącej to dieta zbilansowana, z zaleceniami jak dla każdego innego człowieka	◯	◯
Karmiąc piersią można jeść za dwoje, w dowolnej ilości	◯	◯
Ostre przyprawy w pożywieniu matki powodują kolki u dziecka karmionego piersią	◯	◯
Niektóre substancje smakowe / zapachowe/ inne związki, np. białka przechodzą do mleka matki	◯	◯
Dziecko karmione piersią płacze, bo się nie najada mlekiem matki (pytanie dotyczy w pełni rozwiniętej i ustabilizowanej laktacji)	◯	◯
W czasie laktacji nie wolno spożywać produktów alergizujących, np. cytrusów, czekolady, orzechów	◯	◯
Nasion strączkowe, np. groch, w diecie matkipowodują wzdęcia u dziecka	◯	◯
Dieta eliminacyjna u matki od początku laktacji uchroni dziecko przed problemami z brzuszkiem i alergiami	◯	◯
Dzieci matek, które piją kawę w czasie laktacji często budzą się w nocy	◯	◯
Piwo pobudza laktację	◯	◯
Dieta matki karmiącej to dieta w oparciu o szczególne zalecenia dla kobiet w okresie laktacji	◯	◯
Picie kawy w czasie laktacji jest dozwolone	◯	◯
Dieta eliminacyjna matki karmiącej piersią powinna być włączona tylko z istotnych wskazań medycznych	◯	◯
Mleko matek wegetarianek / weganek jest ubogie w składniki odżywcze, witaminy i minerały	◯	◯
W czasie laktacji wzrasta dobowe zapotrzebowanie kaloryczne i płynowe	◯	◯
Krostki i rumień na buzi niemowlęcia to skaza białkowa - matka musi wyeliminować nabiał z diety	◯	◯
Skład i jakość tłuszczów w mleku matki w dużej mierze zależy od diety	◯	◯
Dieta matki ma wpływ na stężenie jodu w mleku. Stężenie to zależy od zapasów jodu w organizmie matki.	◯	◯
Niedobór witaminy B12 w diecie matki jest czynnikiem ryzyka niedoboru tej witaminy u niemowlęcia.	◯	◯
Dieta matki ma niewielki wpływ na zawartość białka w mleku	◯	◯
Matka karmiąca piersią nie powinna spożywać produktów smażonych i ciężkostrawnych, zalecane są jedynie gotowane i lekkie posiłki	◯	◯

7.05.2020 Dieta matki karmiącej piersią - fakty czy mity?

4. Czy ma Pan /Pani dzieci? Jeśli tak, ile? *

Zaznacz tylko jedną odpowiedź.

- Nie
- 1
- 2
- 3
- 4 lub więcej

5. Płeć *

Zaznacz tylko jedną odpowiedź.

- Kobieta
- Mężczyzna *Przejdź do pytania 17*

6. Jak długo karmiła / karmi Pani piersią pierwsze dziecko? (czas podany w miesiącach) *

Zaznacz tylko jedną odpowiedź.

- nie dotyczy
- 0
- 1
- 2
- 3
- 4
- 5
- 6
- 7
- 8
- 9
- 10
- 11
- 12
- 13
- 14
- 15
- 16
- 17
- 18
- 19
- 20
- 21
- 22
- 23
- 24
- 25
- 26
- 27
- 28
- 29

- 30
- 31
- 32
- 33
- 34
- 35
- 36
- 37
- 38
- 39
- 40
- 41
- 42
- 43
- 44
- 45
- 46
- 47
- 48
- 49
- 50
- ponad 50

7. Jak długo karmiła / karmi Pani piersią drugie dziecko? (czas podany w miesiącach) *

Zaznacz tylko jedną odpowiedź.

- nie dotyczy
- 0
- 1
- 2
- 3
- 4
- 5
- 6
- 7
- 8
- 9
- 10
- 11
- 12
- 13
- 14
- 15
- 16
- 17
- 18
- 19
- 20
- 21
- 22
- 23
- 24
- 25
- 26
- 27
- 28
- 29

- 30
- 31
- 32
- 33
- 34
- 35
- 36
- 37
- 38
- 39
- 40
- 41
- 42
- 43
- 44
- 45
- 46
- 47
- 48
- 49
- 50
- ponad 50

7.05.2020 Dieta matki karmiącej piersią - fakty czy mity?

8. Jak długo karmiła / karmi Pani piersią trzecie dziecko? (czas podany w miesiącach) *

Zaznacz tylko jedną odpowiedź.

- nie dotyczy
- 0
- 1
- 2
- 3
- 4
- 5
- 6
- 7
- 8
- 9
- 10
- 11
- 12
- 13
- 14
- 15
- 16
- 17
- 18
- 19
- 20
- 21
- 22
- 23
- 24
- 25
- 26
- 27
- 28
- 29

- 30
- 31
- 32
- 33
- 34
- 35
- 36
- 37
- 38
- 39
- 40
- 41
- 42
- 43
- 44
- 45
- 46
- 47
- 48
- 49
- 50
- ponad 50

9. Jak długo karmiła / karmi Pani piersią czwarte dziecko? (czas podany w miesiącach) *

Zaznacz tylko jedną odpowiedź.

- nie dotyczy
- 0
- 1
- 2
- 3
- 4
- 5
- 6
- 7
- 8
- 9
- 10
- 11
- 12
- 13
- 14
- 15
- 16
- 17
- 18
- 19
- 20
- 21
- 22
- 23
- 24
- 25
- 26
- 27
- 28
- 29

- 30
- 31
- 32
- 33
- 34
- 35
- 36
- 37
- 38
- 39
- 40
- 41
- 42
- 43
- 44
- 45
- 46
- 47
- 48
- 49
- 50
- ponad 50

10. Jak długo karmiła / karmi Pani piersią najmłodsze dziecko? - proszę odpowiedzieć, jeśli ma Pani więcej niż 4 dzieci; jeśli nie, proszę zaznaczyć "nie dotyczy"(czas podany w miesiącach) *

Zaznacz tylko jedną odpowiedź.

- nie dotyczy
- 0
- 1
- 2
- 3
- 4
- 5
- 6
- 7
- 8
- 9
- 10
- 11
- 12
- 13
- 14
- 15
- 16
- 17
- 18
- 19
- 20
- 21
- 22
- 23
- 24
- 25
- 26
- 27
- 28

- 29
- 30
- 31
- 32
- 33
- 34
- 35
- 36
- 37
- 38
- 39
- 40
- 41
- 42
- 43
- 44
- 45
- 46
- 47
- 48
- 49
- 50
- ponad 50

11. Czy kiedykolwiek przestała Pani karmić piersią i włączyła mleko modyfikowane do diety dziecka z powodu konieczności przestrzegania restrykcyjnej diety eliminacyjnej? *

Zaznacz tylko jedną odpowiedź.

- Tak
- Nie

12. Czy w czasie laktacji stosowała Pani dietę eliminacyjną? *

Zaznacz tylko jedną odpowiedź.

- Tak
- Nie *Przejdź do pytania 17*

13. W czasie karmienia którego dziecka stosowała Pani dietę eliminacyjną? *

Zaznacz wszystkie właściwe odpowiedzi.

- Każdego
- Pierwszego
- Drugiego
- Trzeciego
- Czwartego
- Kolejnego po czwartym

14. Czy dietę eliminacyjną w czasie laktacji zalecił lekarz? *

Zaznacz tylko jedną odpowiedź.

- Tak
- Nie

15. Czy zalecona dieta eliminacyjna wynikała ze wskazań medycznych (np. alergia na białka mleka krowiego)? *

Zaznacz tylko jedną odpowiedź.

- Tak
- Nie

16. Czy dieta eliminacyjna w czasie laktacji wynikała z presji osób w otoczeniu, np. rodziny, znajomych? *

Zaznacz tylko jedną odpowiedź.

- Tak
- Nie

17. Wykonywany zawód: *

Zaznacz tylko jedną odpowiedź.

- Wykonuję zawód medyczny (lekarz, lekarz stomatolog, pielęgniarka, położna, fizjoterapeuta, ratownik medyczny, dietetyk)
- Nie wykonuję zawodu medycznego *Przejdź do pytania 19*

18. Czy kiedykolwiek zalecił Pan / zaleciła Pani matce w okresie laktacji prewencyjną dietę eliminacyjną? *

Zaznacz tylko jedną odpowiedź.

- Tak
- Nie

Metryczka

19. Narodowość: *

20. Miejsce zamieszkania *

Zaznacz tylko jedną odpowiedź.

- Wieś
- Miasto < 100 tys. mieszkańców
- Miasto > 100 tys. mieszkańców

21. Wiek (proszę podać w latach) *

Ta treść nie została utworzona ani zatwierdzona przez Google.

Formularze Google
